# Two-Arm Randomized Pilot Intervention Trial to Decrease Sitting Time and Increase Sit-To-Stand Transitions in Working and Non-Working Older Adults

**DOI:** 10.1371/journal.pone.0145427

**Published:** 2016-01-06

**Authors:** Jacqueline Kerr, Michelle Takemoto, Khalisa Bolling, Andrew Atkin, Jordan Carlson, Dori Rosenberg, Katie Crist, Suneeta Godbole, Brittany Lewars, Claudia Pena, Gina Merchant

**Affiliations:** 1 Department of Family Medicine and Public Health, University of California, San Diego, California, United States of America; 2 UKCRC Centre for Diet and Activity Research (CEDAR), University of Cambridge School of Clinical Medicine, CB2 0QQ, United Kingdom; 3 Children's Mercy Hospital, Kansas City, Missouri, United States of America; 4 Group Health Research Institute, Seattle, Washington, United States of America; National Center of Neurology and Psychiatry, JAPAN

## Abstract

**Background:**

Excessive sitting has been linked to poor health. It is unknown whether reducing total sitting time or increasing brief sit-to-stand transitions is more beneficial. We conducted a randomized pilot study to assess whether it is feasible for working and non-working older adults to reduce these two different behavioral targets.

**Methods:**

Thirty adults (15 workers and 15 non-workers) age 50–70 years were randomized to one of two conditions (a 2-hour reduction in daily sitting or accumulating 30 additional brief sit-to-stand transitions per day). Sitting time, standing time, sit-to-stand transitions and stepping were assessed by a thigh worn inclinometer (activPAL). Participants were assessed for 7 days at baseline and followed while the intervention was delivered (2 weeks). Mixed effects regression analyses adjusted for days within participants, device wear time, and employment status. Time by condition interactions were investigated.

**Results:**

Recruitment, assessments, and intervention delivery were feasible. The ‘reduce sitting’ group reduced their sitting by two hours, the ‘increase sit-to-stand’ group had no change in sitting time (p < .001). The sit-to-stand transition group increased their sit-to-stand transitions, the sitting group did not (p < .001).

**Conclusions:**

This study was the first to demonstrate the feasibility and preliminary efficacy of specific sedentary behavioral goals.

**Trial Registration:**

clinicaltrials.gov NCT02544867

## Introduction

The study of sedentary behavior as an independent risk factor for chronic disease morbidity and mortality has expanded rapidly in recent years [1]. Although historically sedentary behavior has been conceptualized as the absence of physical activity (i.e., physical inactivity) [2], it is now recognized as a distinct behavioral domain, characterized by low energy expenditure (<1.5 METS) and a sitting or reclining posture [3]. An emerging body of epidemiological evidence suggests that sedentary behaviors are associated with all-cause and cardiovascular mortality, overweight and obesity, type 2 diabetes, depression and psychological well-being [4–12]. Importantly, many of these associations were observed independent of participation in moderate to vigorous intensity physical activity. Further, there is preliminary evidence that the physiological mechanisms through which sedentary behavior negatively impacts health are distinct from the pathways linked to physical activity [13,14].

Surveillance data indicates that adults accumulate over 7 hours per day of sedentary time [15], yet the ‘dose’ of sedentary behavior that most accurately predicts health risk remains uncertain. There are multiple ways of disrupting sedentary time such as through movement, prolonged standing, or brief sit-to-stand transitions. Approaches for disrupting sedentary time likely vary in terms of their acceptability by different populations and may have differential impacts on health outcomes.

A recent review suggests that physical activity interventions do not appreciably alter sedentary time [16]. Thus, there are an emerging number of interventions that explicitly aim to reduce sedentary behavior. The majority of interventions to date have targeted children or working adults. Among the adult studies, the workplace has been the primary setting and interventions have centered on environmental changes including implementing standing desks to promote less sitting [17,18]. These interventions have shown some efficacy but may not be appropriate in nonworking populations, such as retired older adults. Further, most of the interventions have delivered mixed messages encouraging breaks from sitting, prolonged standing, and movement, making it difficult to determine which types of sedentary behavior are most feasible to improve and which types of intervention most effective. Most studies have observed reductions in sitting time and increases in standing time with little impact on sit-to-stand transitions [17,18]. However, prolonged standing may not be feasible or safe for some populations (e.g., older adults), may not be convenient outside of workplaces, and may be more difficult to maintain long term compared to other approaches such as brief sit-to-stand transitions. Laboratory studies suggest that the frequency of disrupting sitting is important, and that sit-to-stand transitions can increase postural blood flow, contract muscles, and stimulate biological processes important in disease formation [19–22].

Therefore, we developed two different intervention approaches for sedentary behavior reduction: 1) reducing overall sitting time and 2) frequent brief sit-to-stand transitions. Working and non-working adults aged 50–70 were randomized to each condition to examine the feasibility of each target and preliminary efficacy on objectively measured sedentary behaviors and physical activity. We hypothesized that each intervention would only affect the behavior targeted by the intervention content.

## Materials and Methods

S1 CONSORT Checklist outlines the CONSORT 2010 checklist for randomized trials and where the information is included in the manuscript.

### Participants and Recruitment

We conducted a two arm randomized pilot trial with an equal number of workers (employed full time) and non-workers assigned to each condition. This was to ensure variability in work status, not to explore differences by work status. The study was supported through a small departmental grant to determine the acceptability and feasibility of a sedentary behavior intervention. Therefore, the sample size and intervention were designed to maximize funds and test the intervention in a small sample of 30 participants, as appropriate for a pilot trial. Increased power to detect differences was also derived from the continuous data collection of the primary outcome across multiple days per participant.

Participants for the study were recruited starting in September 2013 and the final participant was seen in March 2014. Working participants were recruited to the ‘Take a Stand’ study through a flyer posted on a university listserv as well as word of mouth. Non-working participants from the local community responded to an online advertisement. Interested individuals called a study phone line and were screened for eligibility by a research assistant. Participants were eligible if they were aged 50–70 years, spent at least 8 hours per day sitting on average over 5 days (assessed by ActivPAL), were able to attend 4 measurement visits with study staff in 4 consecutive weeks, were willing and able to wear a thigh mounted inclinometer 24 hours per day for 21 days (including the baseline monitoring week), were able to read and write in English, were able to provide written informed consent and did not have a serious chronic condition that would limit their ability to stand. Eligible participants came to the study office and were provided further information on the study and completed a written informed consent. Once participants signed the consent form, they wore a thigh mounted inclinometer for 7 days to determine whether they met the 8 hour minimum sitting time eligibility criteria. 8 hours was selected as it was above the US national average for sitting time (15) and a 2 hour reduction was possible. Participants returned to the study office and, if they met the study criteria, were randomized to one of the two study conditions using a randomization table developed by a statistician. The table included 30 spots with masked group assignments and was password protected. The study coordinator added new participants to the table in the order in which they were enrolled and unmasked their group assignment. The participant’s assignment was given to study personnel who then notified the participant. Once 15 workers had been enrolled, only non-workers were screened for eligibility. The statistician was blinded to the intervention assignment. Data collection staff were not as they had to select the correct algorithm to process and visualize the data, according to the specific intervention. The data were then given to the health educator to provide in person feedback to the participant each week. Because the data were objectively collected with the ActivPAL we believed measured bias was limited. The study was registered with clinicaltrials.gov following study completion with results. All study activities were approved by the University of California, San Diego institutional review board on July 18^th^, 2013. The authors confirm that all ongoing and related trials for this intervention are registered. The research protocol and consent documents can be found in the supplementary information (S1 Protocol).

## Procedures

Following randomization, participants answered a short written survey, completed an in person interview about the feasibility and acceptability of wearing the thigh mounted inclinometer and met with the study health educator for one hour for their first intervention session. Participants then returned to the study office one and two weeks later to have data from their inclinometer downloaded by staff, have a follow-up session with the health educator, and complete study surveys and interview questions.

### Intervention Conditions

Participants were randomized to either reduce their total sitting time or increase sit-to-stand transitions. Each intervention lasted 14 days with three in person health educator sessions during that time. Both intervention arms drew from behavior change strategies highlighted as effective, including, self-monitoring, goal setting, feedback, problem-solving, and planning [23]. In addition, the intervention was informed by the social ecological model [24,25] that considers the supportiveness of the physical and social environments in which behaviors occur. Information was provided in person, through written materials and by emails and phone calls in both conditions. Both groups received written educational materials on the dangers of excessive sitting and reviewed a generic day to illustrate how many sitting opportunities individuals face each day. During each session, the health educator also discussed the benefits of sitting less or increasing sit-to-stand transitions (depending on study condition) and brainstormed potential barriers to implementing the new behavior as well as strategies to overcome these barriers. At the end of each session, participants completed and signed a behavioral contract that outlined the goal for the upcoming week, their motivation to achieve the goal, and a potential barrier and solution to implementing the behavior. Participants created an action plan with the health educator indicating how they would incorporate study tools to accomplish the goal during the upcoming week. At the end of the visit, participants rated their confidence in meeting the goal on a scale from 1 (not at all confident) to 10 (completely confident). If participants rated their confidence at a 5 or below, the health educator would revisit the action plan to develop different strategies to help the participant feel more confident. Participants selected whether they wanted email or phone call check-ins in the second week of the intervention to test whether this mode of communication was also useful.

#### Reduce sitting condition

Those randomized to this condition focused on reducing their overall sitting time by two hours per day (a goal achieved in similar studies [17,18] that represented approximately a 25% reduction in daily sitting time). Participants were encouraged to reach this goal by standing in bouts of roughly 10 minutes per hour. The purpose of this arm was to investigate whether we could replicate improvements in sitting time achieved in other worksite studies in our cohort of older adults, which included both workers and non-workers.

During weekly meetings with the health educator, participants in this group reviewed feedback charts depicting their sitting and standing time, as measured by the thigh worn inclinometer, across each day of the previous week. They discussed when they might be able to reduce their sitting during each day, how to incorporate extended standing breaks into their working and home lives, how to set up a social and physical environment to support this goal, and how to track their progress towards the goal over time. Participants were provided with a choice of tools to support their behavior change including: standing desks, timers (e.g., phone apps, computer apps), physical timers that could be placed in a variety of locations (e.g., work desk, on top of TV, kitchen counter), a vibrating watch, a branded study bracelet with the study tagline to serve as a reminder, and texts, emails, or phone calls from study personnel.

#### Increase sit-to-stand transitions condition

Those randomized to the sit-to-stand condition focused on increasing the number of sit-to-stand transitions they performed throughout the day with a goal of adding 30 additional transitions per day. Previous studies have not succeeded in increasing the number of sit-to-stand transitions in older adults, possibly because they focused on reducing overall sitting time, encouraged longer standing breaks and did not provide a specific goal for sit-to-stand transitions [26–28]. An increase in sit-to-stand transitions would not be expected with an increase standing intervention alone, as prolonged standing reduces the opportunity for sit-to-stand transitions.

During each weekly session with the health educator, participants in this group reviewed a feedback chart illustrating the number of sit-to-stand transitions they achieved each day, measured by the thigh worn inclinometer. Participants were told that each standing break could be brief and did not have to interrupt normal activities. Thus, we felt that social or environmental supports were not required but that more frequent cues to remind participants to stand up would be more useful. Participants were provided with the same choice of reminder tools to support their behavior change as the ‘reduce sitting group’ except they were not given standing desks. Instead, they were offered counters to help them track sit-to-stand transitions throughout the day (e.g., electronic counter, bracelet counter, dry erase board). The intervention materials and protocol are available on request from the authors.

#### In person interviews

At each visit, participants completed a short semi-structured interview with study staff. They were asked about the acceptability of the study device, their satisfaction with the intervention materials, and the feasibility of the intervention goals. Questions probed the experience of wearing the device, their success following the action plan, barriers and facilitators to behavior change, the impact on other behaviors, and their goal progress. Responses to these questions were deemed secondary outcomes.

### Primary Outcome: Objective Measures of Behavior

The thigh worn inclinometer (the activPAL3, PAL Technologies Limited, Glasgow, UK) was employed as an intervention feedback tool as described above and provided the outcome measures, which included: daily sitting time, daily standing time, daily stepping time and number of sit-to-stand transitions per day. Participants completed a sleep log and daily waking hours were extracted to omit sleep time from the measures. Since sleep time can greatly influence the number of waking hours available for sitting, it was important to analyze sitting time reductions irrespective of sleep time. Participants were shown how to attach the inclinometer to their mid-thigh and to waterproof the device with an adhesive surgical sleeve provided by study staff. Replacement sleeves were provided, but participants were encouraged not to remove the device between study visits as the waterproofing allowed it to be worn during showering. At each office visit, the device and covering were replaced.

### Analyses

All participants were analyzed according to the intention to treat (i.e. the condition to which they were assigned). There was no missing data as all participants were compliant with the 21 day wear protocol. Statistical analyses were performed with SPSS v22. The four outcomes (sitting time, standing time, stepping time, and number of sit-to-stand transitions) were analyzed for their trajectories over time. Mixed effects regression analyses, with days nested within participants, were performed for each outcome separately. A time by condition interaction was investigated. Work status was entered as a covariate. The number of sit-to-stand transitions was natural log transformed. All analyses adjusted for activPAL wear time.

No formal analysis of the secondary interview data was performed. We merely report here information that helps to explain the study findings and provide evidence of feasibility and acceptability.

## Results

**Fig 1:**
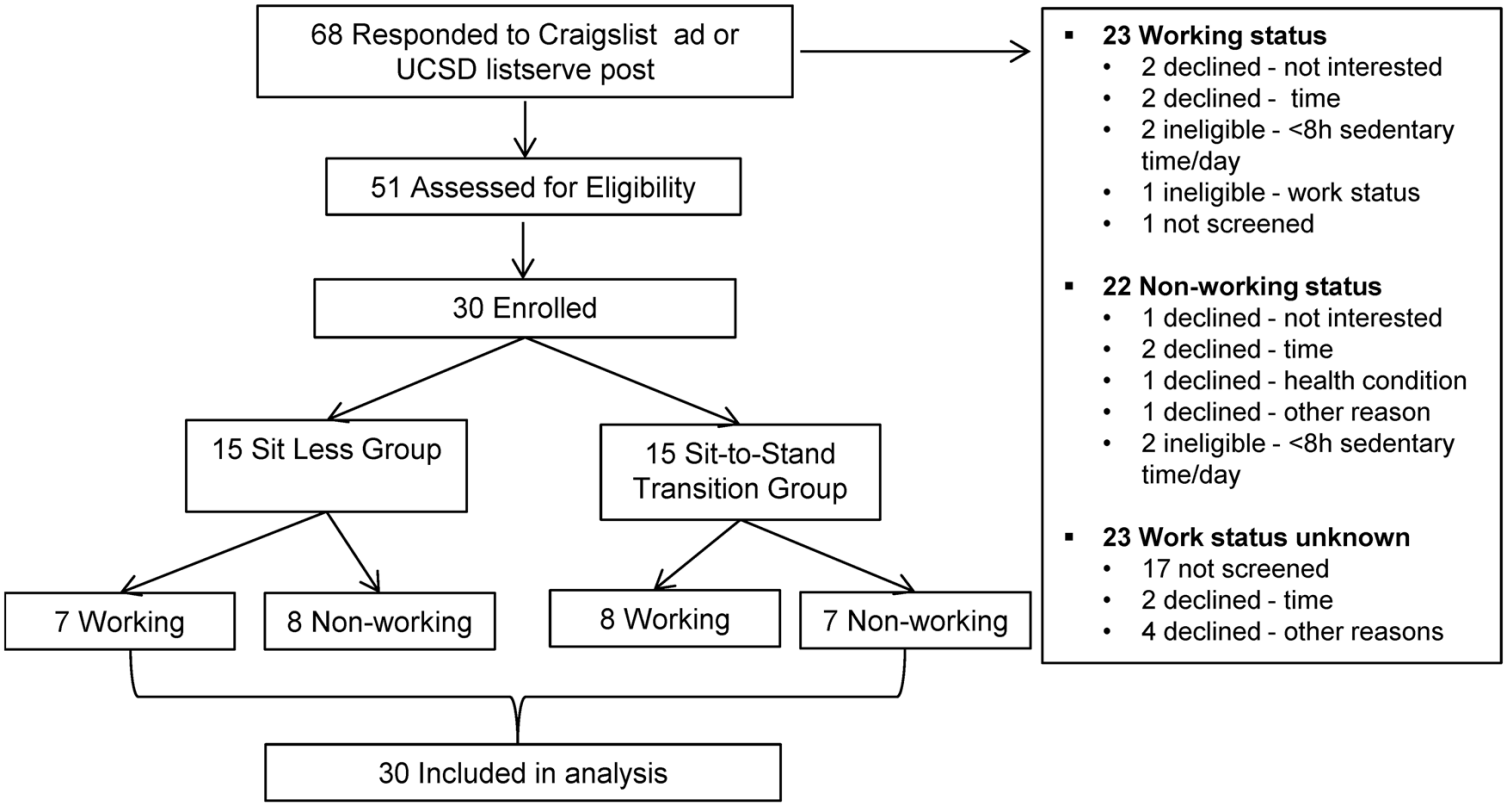
Flow diagram of progress through randomized pilot study.

Fig 1 provides a CONSORT diagram of recruitment for the pilot intervention. A total of 51 people were screened for participation; 80% were eligible. Four participants were ineligible because they did not meet the minimum average 8 hour/day sitting time criteria during the enrollment week and 2 participants were ineligible based on work status (i.e., full-time employed after the working group was closed to enrollment). A total of 15 participants declined participation based on a health condition precluding participation, lack of interest in the study, and/or lack of time to attend study visits. Table 1 highlights the demographic characteristics of the two intervention groups. The average age was 60.4 years old (SD 5.9), the average body mass index was 27.0 (SD 4.7). By design, half the participants were employed, 80% were white, 57% were married and 27% were male. All participants enrolled into the study completed the study, all health education sessions were delivered as anticipated, and all participants complied with activPAL wear time expectations (24 hours for 21days). No adverse events were reported during the intervention period.

**Table 1:** Demographic characteristics (N = 30).

	Mean (SD)/Frequency (%)		
Demographic Variables	Reduced Sitting Group (n = 15)	Sit-to-Stand Transition Group (n = 15)	Total
Age	61.0 (6.0)	60 (6.0)	60.4 (5.9)
Anthropometrics			
Body Mass Index (m^2^/kg)	26.2 (4.7)	27.7 (4.8)	27.0 (4.7)
Work Status			
Full-Time Employed	7 (46.7)	8 (53.5)	15 (50)
Not Full-Time Employed	8 (53.5)	7 (46.7)	15 (50)
Gender			
Female	11 (73)	11(73)	22 (73)
Male	4 (27)	4 (27)	8 (27)
Race/Ethnicity			
White non-Hispanic	14 (93.3)	10 (66.7)	24 (80)
All other	1 (6.7)	5 (33.3)	6 (20)
Marital Status			
Married	7 (46.7)	10 (66.7)	17 (57)
Not Married	8 (53.3)	5 (33.3)	13 (43)

SD = standard deviation

### ActivPAL data

**Fig 2:**
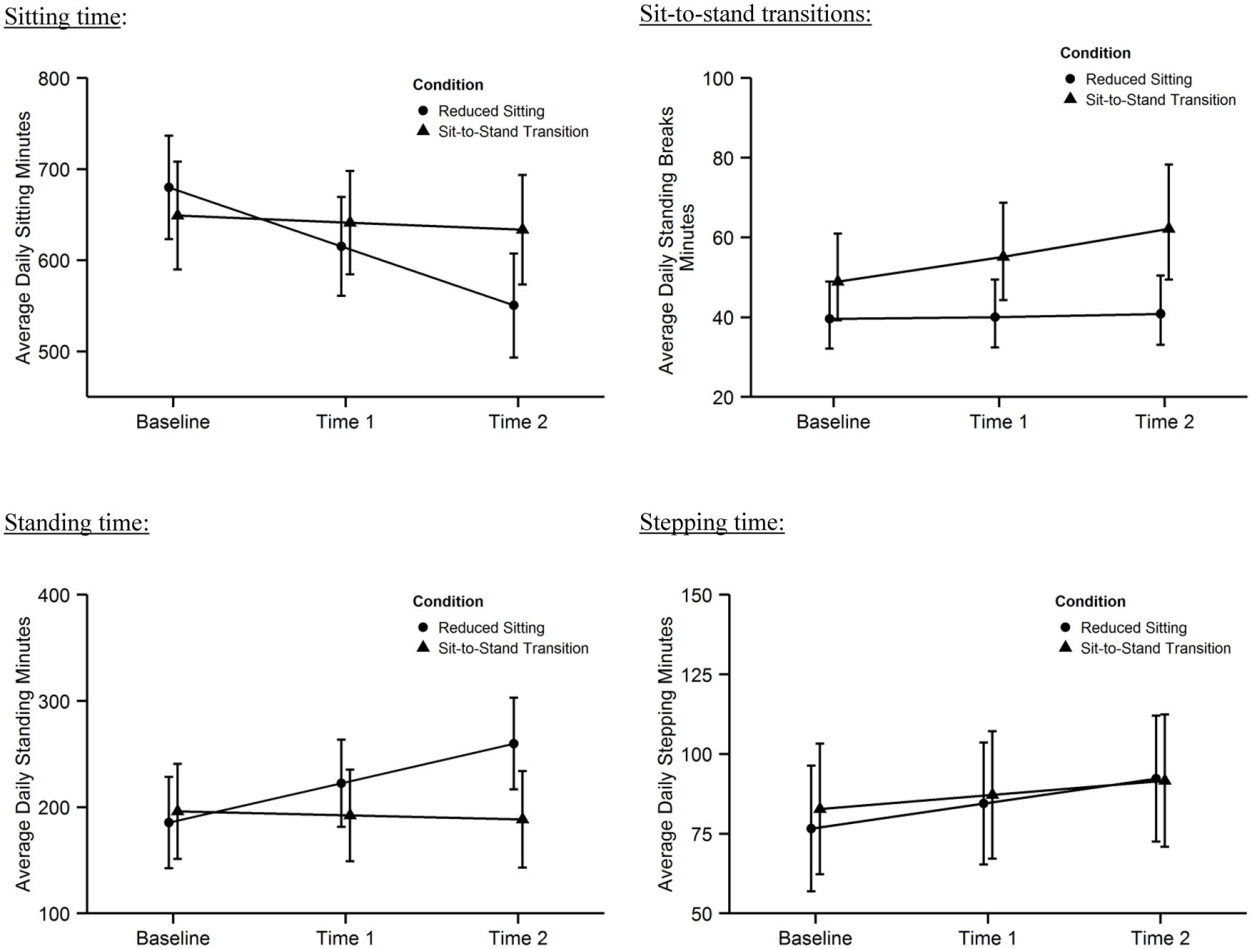
Changes in daily sitting, standing, sit-to-stand transitions and stepping over time by condition.

Analyses were performed on 21 days of data for 15 participants in each arm. Fig 2 shows the changes over time by condition for all the outcomes, regardless of their significance. There was a significant time x condition interaction for sitting time (Beta 57.0 (SE 12.5): p < .0001), standing time (Beta -40.9 (SE 9.4): p < .0001), and number of sit-to-stand transitions (0.10 (0.04): p = .006). The interaction term describes the difference in change between groups over time in minutes or transitions per day. Those randomized to reduce their sitting time had a significant 130 minute decrease in sitting time but no change in sit-to-stand transitions. Those randomized to increase sit-to-stand transitions had a significant increase in sit-to-stand transitions by about 13 transitions per day, but no change in total sitting time. There was no significant interaction for stepping time; all participants increased their physical activity level by 10 minutes/day. Table 2 presents the full results of the statistical models, including 95% confidence intervals to inform future interventions.

**Table 2:** Results of statistical models, adjusting for employment status.

	Sitting Time		Standing Time		Stepping Time		Sit-to-Stand Transitions	
	β	95% CI	β	95% CI	β	95% CI	exp(β)	95% CI
Intercept	679.86	(623.02, 736.7)	185.42	(142.44, 228.39)	76.65	(56.95, 96.35)	39.5	(31.89, 48.93)
Sit-to-Stand Transitions Group	-30.91	(-99.28, 37.47)	10.6	(-41.09, 62.29)	6.1	(-17.47, 29.67)	1.24	(0.96, 1.6)
Reduced Sitting Group (Ref)								
Time*	-64.76	(-81.79, -47.74)	37.19	(24.34, 50.04)	7.81	(2.95, 12.68)	1.02	(0.97, 1.07)
Sit-to-Stand Transitions Group X Time	57.02	(32.49, 81.55)	-40.98	(-59.5, -22.47)	-3.38	(-10.39, 3.63)	1.11	(1.03, 1.19)
Reduced Sitting Group X Time (Ref)								
Full-Time Employed	-76.23	(-140.47, -11.99)	71.9	(23.33, 120.48)	24.14	(1.54, 46.74)	1.06	(0.83, 1.35)

* Refers to study time points (baseline, Time1, and Time 2)

CI = confidence interval

Ref = reference group

Data were available for a subset of participants (N = 22) processed with the sit-to-stand transition detection every second. The default setting is that only stands of 10 seconds are recorded. Our participants were not instructed to stand for that long. Data from the subset indicated that the average increase in stands achieved in the sit-to-stand group was 40. This demonstrates that participants met their goal of 30 additional sit-to-stand per day.

### Interview data

The in-person interviews revealed that the intervention was acceptable and feasible. Participants were comfortable wearing the thigh worn inclinometer 24 hours a day for 21 days. Participants understood their behavioral goals and found the information presented to them from the graphical data helpful. They were also satisfied with the modes of intervention delivery and intervention content.

Participants chose from a range of tools to help them. Most participants did not recognize how challenging the behavior change would be and selected few supportive tools in the first week. In the second week, they were more cognizant of the barriers and selected more tools to support their behavior. The barriers that participants faced were context specific and varied by personal situations and day-to-day routines. This suggests that individualized support is required, at least in the early stages of behavior change. Participants found regular cues to behavior change helpful, e.g. a phone alert, but they had to remember to set the reminder. Participants reported difficulty implementing the behavior on weekends because of the variability in activities they did compared to the weekdays. It was challenging to integrate the targeted behavior change into activities that were outside their daily routines.

During the interviews, participants reported a variety of benefits associated with either sitting less or increasing sit-to-stand transitions. For example, several participants mentioned drinking more water while working towards the goal because standing provided more opportunities to move around the home or office. Other participants mentioned more social interactions at work as they would opt to walk to a co-worker’s office to talk, as opposed to sending an email or using the phone. Additionally, participants also indicated that they accomplished more household chores during the study and were more productive in the evenings based on their motivation to achieve the study goals.

## Discussion

This was the first randomized control trial of two different strategies to interrupt sitting conducted in older adults. Recruitment, measurement and intervention delivery all proved feasible and acceptable. The results showed that each intervention group focused on the behavior goal they were given and only the targeted behavior changed. For example the reduce sitting time group decreased their daily sitting time on average by the recommended two hours (from 644 minutes to 514 per day) and increased their standing time, but their sit-to-stand transitions did not change. The sit-to-stand transition group increased their transitions (on average 10 extra transitions per day), but did not decrease their daily sitting time. These findings support the paradigm that reducing sitting and increasing sit-to-stand transitions are independent behaviors and require distinct and specific goals. Although there was a significant increase in physical activity over time in both groups, the increase in minutes was small and was not sufficient to meet physical activity recommendations. This lends support to the idea that sedentary behavior is independent of meeting daily physical activity guidelines.

The increase in sit-to-stand transitions, although significant, was smaller than the 30 per day increase encouraged by the health educators. The overall smaller success in this target behavior could have been due to the inclinometer default setting which only registered breaks lasting at least 10 seconds; thus a sit-to-stand transition that involved less than 10 seconds of standing would not have been captured by the measurement device. Part way through the intervention we discovered this default could be changed and analyses in a subsample of participants indicated average increases of 40 stands per days. The eligibility criterion for the study was at least 8 hours of sitting on average, per day. There was no criterion related to the number of sit-to-stand transitions participants took at baseline. Therefore, some participants already had high numbers of sit-to-stand transitions at baseline, despite also meeting the daily 8 hour per day sitting time criteria. Achieving the goal of 30 extra stands throughout the day was perhaps more difficult in these individuals. In future studies, eligibility criteria should also preclude participants with a high number of sit-to-stand transitions.

Previous studies have shown similar reductions in sitting time and increases in standing time as seen in our ‘reduce sitting by two hours’ condition [17,18]. No previous study has targeted sit-to-stand transitions with a specific goal (e.g. 30 additional transitions); although some studies did encourage frequent breaks [17,27] and only one previous study has achieved a significant change in this behavior but only increased transitions by about an additional 4 breaks per day [26,28]. Other studies have shown no change in physical activity [17], or small changes as seen in our study [27]. Our study adds to this body of literature because few previous sedentary behavior intervention pilot studies have utilized a randomized design [29–32]. Furthermore, most studies did not include participants over 65 years of age and were delivered exclusively in worksite settings [17,29]. Three previous studies in older adults used only pre-post designs and did not find as large a reduction in sitting as in our cohort [26–28].

Our study strengths include the randomized group comparison, objective measures of behavior, inclusion of older adults, and a working and non-working population. Limitations include the short intervention period and predominantly educated and white participant group. Further, study staff were not blinded to the intervention because it involved weekly feedback to participants.

## Conclusions

This is the first pilot study in older adult workers and non-workers to attempt to interrupt sitting behaviors in two different ways. One strategy was to reduce sitting time by 2 hours per day; the other was to increase daily sit-to-stand transitions by 30. Both groups changed the targeted behavior exclusively without changing the other behavior. Participants were compliant and satisfied with the intervention and measurement procedures. Future studies can build on our intervention procedures by testing the intervention strategies in larger samples for longer periods and adding health outcomes.

## Supporting Information

S1 CONSORT Checklist CONSORT 2010 checklist of information to include when reporting a randomized trial. (DOCX)

S1 Protocol Research protocol and consent documents (PDF)

## Abbreviations

SD: standard deviation
SE: standard error
