# Supplementary material for: Two-Arm Randomized Pilot Intervention Trial to Decrease Sitting Time and Increase Sit-To-Stand Transitions in Working and Non-Working Older Adults
Source: PLoS One. 2016 Jan 6;11(1):e0145427. doi: 10.1371/journal.pone.0145427 (PMC4703201; doi:10.1371/journal.pone.0145427)
Supplement: S1 Protocol — (PDF) [file pone.0145427.s002.pdf]

UCSD Human Research Protections Program
New Biomedical Application
RESEARCH PLAN

Instructions for completing the Research Plan are available on the HRPP website.
The headings on this set of instructions correspond to the headings of the Research Plan.
General Instructions: Enter a response for all topic headings.
Enter “Not Applicable” rather than leaving an item blank if the item does not apply to this project.

Version date: 05/11/2011

1. PROJECT TITLE

HRPP Project #130817 - Optimizing sedentary behavior interventions to affect acute physiological changes

2. PRINCIPAL INVESTIGATOR

Jacqueline Kerr, PhD

3. FACILITIES

UCSD Atkinson Hall – 3rd floor

4. ESTIMATED DURATION OF THE STUDY

8 months (September 2013- April 2013)

5. LAY LANGUAGE SUMMARY OR SYNOPSIS (no more than one paragraph)

An emerging body of epidemiological evidence suggests that various forms of sedentary behavior, including TV viewing, occupational sitting, and total daily sitting, may be associated with all-cause and cardiovascular mortality, overweight and obesity, type 2 diabetes, depression and psychological well-being. Importantly, many of these associations were independent of participation in moderate to vigorous intensity physical activity. We propose a pilot study to assess the feasibility, acceptability and preliminary efficacy of two interventions targeting sedentary behavior. Since it is currently unknown what component of sedentary behavior exposure presents the greatest risk to health, we propose separate interventions to reduce overall sedentary time and to promote breaks in sedentary time.

6. SPECIFIC AIMS

Primary aim: To determine the acceptability and feasibility of selected personal, social and environmental strategies to reduce overall sitting time and increase the number of times participants stand up in a day.
Secondary aim: To assess whether existing and new measurement approaches can detect specific changes in sedentary behavior.
Exploratory aim: To establish whether specified intervention strategies were efficacious in reducing sedentary behavior and whether intervention effects were specific to the targeted sedentary behavior construct (e.g. decreased overall sitting time or increased number of breaks in sitting).

7. BACKGROUND AND SIGNIFICANCE

The study of sedentary behavior as an independent risk factor for chronic disease morbidity and mortality has expanded rapidly in recent years [1]. Historically, sedentary behaviors were conceptualized as the absence of physical activity [2]. Today, however, they are recognized as a distinct domain of behavior, characterized jointly by low energy expenditure (<1.5 METS) and a sitting or reclining posture [3]. This group of behaviors, which take place in occupational, transport, leisure and domestic contexts, are highly prevalent in developed countries. Surveillance data from the USA, for example, indicates that adults accumulate over 7 hours / day of sedentary behavior [4]. An emerging body of epidemiological evidence suggests that various forms of sedentary behavior, including TV viewing, occupational sitting, and total daily sitting, may be associated with all-cause and cardiovascular mortality, overweight and obesity, type 2 diabetes, depression and psychological well-being [5-12]. Importantly, many of these associations were independent of participation in moderate to vigorous intensity physical activity. Concurrently, laboratory-based studies have provided preliminary evidence of unique physiological pathways that may account for these adverse relationships; processes that are distinct from the physiology of physical activity [13, 14]. Considered alongside high prevalence rates, the potential population health burden of sedentary behavior is considerable. However, the ‘dose’ of sedentary behavior that most accurately predicts health risk remains uncertain. It is unclear, for example, whether adverse associations with health are driven by engagement in specific behaviors (e.g. TV viewing), by the overall volume of accumulated sedentary time, or by a particular pattern of accumulation (e.g. prolonged bouts with few breaks).

The development and application of precise measures of sedentary behavior is a key challenge in this field [15, 16].

The tools applied currently in population-based studies of sedentary behavior typically capture only one feature of the sedentary behavior construct (energy expenditure *or* posture) or infer sedentariness from self-reported behavior (e.g. TV viewing). Self-report assessments have been used most frequently to date but these are susceptible to various forms of bias (i.e. recall bias / social desirability). Improved methods of measurement are required to establish the exposure that presents greatest risk to health and for the evaluation of interventions aimed at modifying sedentary behavior. In this latter case, instruments that are specific to the intervention outcomes and that demonstrate responsiveness (i.e. the ability to detect *change* in the outcome) are essential.

Behavior change interventions typically seek to influence the modifiable antecedents (correlates / determinants) of the target behavior [17, 18]. Although research in this area is relatively limited, it appears that the determinants of sedentary behavior are distinct from those of physical activity [19]. In addition, the cognitive, behavioral, contextual and environmental characteristics of sedentary behaviors are such that an alternative approach to the design of intervention programs may be warranted. There is good evidence that much of human behavior occurs without conscious reflection or decision making but rather is automatic, cued by features of the social, temporal or physical environment [20]. Habits are one such example of this process in action – habitual behaviors are cued by external stimuli without necessary reflection on the goal of that behavior. Many sedentary behaviors may operate in this way; behavioral patterns that are established through routine and cued by familiar social and physical environments that promote sitting [21]. Traditional self-monitoring methods may not be effective for a behavior that spans the whole day, because very frequent monitoring is required which may quickly become tedious. At present, there are relatively few published interventions targeting sedentary behavior in adults, and most existing studies focus upon reducing leisure-time behaviors (e.g. TV viewing) through strategies targeting conscious decision making [22]. Some physical activity studies have been shown to reduce sedentary behavior, but many have not suggesting that a more specific approach may be required [23].

We propose a pilot study to assess the feasibility, acceptability and preliminary efficacy of interventions targeting sedentary behavior. Since it is currently unknown what component of sedentary behavior exposure presents the greatest risk to health, we propose separate interventions to reduce overall sedentary time and promote breaks in sedentary time (sit to stand transitions). Studies of the relationship between health and sedentary behavior focus on these components (usually not in the same study), but it is not clear which may have the greatest effect on biological/physiological mechanisms and which is likely to be the most effective strategy to change sedentary behavior. We will explore the utility of a range of behavior change strategies that focus upon modifying personal, social and environmental cues to behavior. Ecological models of behavior change and Social Cognitive Theory both emphasize the importance of environmental cues and in the case of sedentary behavior the ubiquitous presence of chairs is a barrier to reducing sitting time. Standing desks (which are available in many low costs formats) have emerged as the number one environmental manipulation for reducing sitting time in the occupational setting [24, 25]. Social norms related to sitting may also be a barrier to increasing standing time. Addressing social norms through public declarations of intentions to stand during meetings, for example, is in line with many theories of behavior change (e.g. the Theory of Planned Behavior) [21]. Finally, because sitting is an automatic behavior that dominates our daily habits, real time decision prompts are required to remind us how long we have been sitting and to cue a decision to take a standing break. Theories of habit modification support such triggers, as does the work of BJ Fogg on persuasion, behavior triggers and changing 'tiny' habits [26]

8. PROGRESS REPORT

N/A

9. RESEARCH DESIGN AND METHODS

Study design: A two arm randomized design will be used. All participants will undergo a 1 week monitoring period to assess sedentary behavior at baseline. Participants who sit for at least 8 hours per day will be randomized to one of two possible conditions for a 2-week intervention. The intervention arms will focus on 2 different sedentary behavior goals: 1) reducing overall sedentary time, or 2) increasing breaks in sedentary time (i.e. sit to stand transitions). In order to establish the most feasible and acceptable means of modifying sedentary behavior, three behavior change strategies will be tested within each intervention arm: 1) personal cues (e.g. self-monitoring / timed prompts), 2) social cues (e.g. walking meetings, behavioral contracting), 3) environmental cues (e.g. standing desks, TV time managers).

Older adults, aged 50-70, both working and retired, will be recruited. Older adults are the most sedentary population group who could experience meaningful health benefits from reducing sitting time and increase sit to stand transitions.

They are also a group who may find the transition to physical activity challenging, but a reducing in sedentary time could provide a more gentle transition to greater movement. Further understanding the transition from working adult to retired older adult is an important life change with health consequences. We will be able to study behaviors at work and home to assess the challenges of these different environments in reducing sitting time. People interested in participating in the study will call our offices to be assessed for eligibility. Verbal consent to assess eligibility will be obtained, as this information poses minimal risk. Written consent will be obtained prior to beginning the research study at the first office visit.

Eligible participants will attend a total of 4 visits at the UCSD offices that will last approximately 60-90 minutes each. In the first visit, participants will meet with research staff to receive a more in-depth orientation to the study protocol and provide informed consent. After consent is obtained, participants will learn how to wear the study sensor (device described below). They will return to the offices 1 week later and will receive a standard educational introduction to sedentary behavior and complete a short survey about their usual habits. The first week of wear will serve as an informal “run in” period to ensure compliance with the device wear as well as to determine that the participant has at least 8 hours of sitting time per day. The study is dependent on the collection of data and thus their participation would be rendered useless without this information. Additionally, the intervention is aimed at reducing sedentary time, thus those with active jobs or lifestyles will not be eligible to continue in the study. Participants with <8 hours of sitting time in the baseline week would receive $25 for wearing the devices but will be removed from the study.

Participants will then receive instructions & materials specific to the condition to which they were randomly assigned. The health coach will print a graph that illustrates the times of day that participants were sitting during the previous week. They will then brainstorm with the participant about how they think they might be able to reduce those extended bouts of sitting. Multiple tools and strategies will be presented. Those in the intervention designed to reduce overall sitting time will be offered the use of a standing desk for the 2 week intervention. Our staff will install this in their office. Both groups will be offered alarms, timers, and counting devices to help them track the number of times they take standing breaks. Logs will be provided for them to record these breaks throughout the day. Participants will choose which strategies they’d like to try in week 1. They will return 1 week later to complete an interview about their experience that week and meet with the health educator about their progress. Data from the measurement device (activPAL™) will be downloaded and graphical feedback will again be provided to assess whether they met their goals.
Participants will complete simple daily logs that track when they go to bed, wake up, and arrive and leave work so that we can provide better feedback on intervention behaviors, (i.e. we will know if someone is sleeping at night versus sitting for a prolonged period watching TV). Alternative tools and strategies will be discussed if the participant needs further support in week 2 to meet the intervention goals. Participants will return for their final visit1 week later to complete an exit interview with study staff and a short survey. Participants will be contacted by staff each week to ensure they are not experiencing any problems with the devices or to answer any questions. They will also select their preference for either a text, email or telephone reminder relevant to their intervention condition. For this pilot work, we will employ the most feasible low cost intervention strategies available, for example existing phone apps or mechanical reminders such as alarms and self-constructed standing desks. In the future grants, we will develop more custom built technological solutions and will explore features of these in the proposed interviews.

<u>Participants</u>: A sample of 30 working and non-working, older adults will be recruited from the University campus as well as the surrounding community. In an effort to maximize variability in the population, we will attempt to recruit a sample that includes at least 10 Hispanic participants and 10 overweight to obese participants. Future grants will investigate whether biological and physiological mechanisms function similarly in obese and non-obese populations. We do not plan to explore this in the pilot data but need to understand whether barriers to recruitment and intervention are different by weight status and ethnicity.

Study visits will take place in the Family and Preventive Medicine offices in on the 3rd floor of Atkinson Hall at UCSD or in Suite B122 at the La Jolla Village Professional Center in La Jolla. Parking will be provided.

Study Timeline

Visit 1	Consent and device distribution	

Visit 2	Sedentary behavior introduction, randomization to study intervention and instructions, complete survey, interview, continue to wear devices	$50 ($25 if not eligible: they do not stay for survey & introduction)
Visit 3	Interview, meet with Health Educator, complete survey, continue to wear devices	$50
Visit 4	Interview, meet with Health Educator, complete survey, return devices.	$50

Measures:

Sedentary behavior will be measured objectively with the activPAL™ (PAL Technologies, Ltd). The activPAL™ is currently the gold standard for measuring sitting time and sit to stand transitions. Participants will be asked to wear the device for 24 hours per day, 7 days per week for the duration of the 3 week study. This is a small (measuring 53 x 35 x 7mm) and lightweight (15 grams) device that measures movement. The activPAL is worn on the thigh using a mild adhesive. These monitors have been effectively used with older adult populations; however it is unknown whether obese older adults will be willing and able to wear them. Part of our study includes getting feedback, and we will make it clear to participants they can stop wearing the devices at any time. Research staff will fit participants with an activPAL monitor at their baseline measurement visit and provide them with written instructions to reapply the activPAL at home, if necessary. Instructions to waterproof the devices will be provided so that they may be worn when showering, bathing or swimming. At each office visit, the device will be removed for data retrieval and charging. Participants will wear the device continually for the three weeks of the study. RAs will upload participants' activity data and process it into a graph that will be used by the health educator to help with goal setting and overcoming barriers to reducing sedentary time.

Outcomes: The primary outcomes for the pilot studies will be acceptability and feasibility assessed by questionnaire and semi-structured interviews, conducted at the end of each study week. Feasibility assessments will target (i) participant recruitment, (ii) strategy compliance, and (iii) overall engagement with the suite of techniques. A questionnaire will examine participants overall satisfaction with intervention content and delivery, their ranking of the various interventions that were employed (both intervention goals and intervention strategies), and compliance with intervention protocols. Participants will also be asked to log how frequently and for how many days they employed the intervention strategies to inform compliance. Acceptability and compliance with wear time requirements of the objective measurement sensors will also be assessed. The Semi-structured interviews will be conducted to ascertain participants' perspectives on various aspects of intervention goals, content, delivery, barriers and facilitators to success, context and recommendations for future studies. These interviews will be recorded and transcribed by RAs. At the end of the study, we will also discuss alternative intervention tools and goals and prompt them to choose which strategies they would prefer.

Sample questions include:

- Please tell us about wearing the device this week? How did it fit into your daily routine?
  - What sorts of issues, if any, did you have with the device?
- Tell us about taking the breaks from sitting. How did they fit into your daily routine?
  - How did you feel about the number of breaks you were asked to take? Can you tell us how you managed or did not manage to take all of the breaks?
  - What sorts of things did you do while taking a break?
- You received prompts to remember to take sitting breaks. In what ways were these reminders helpful or not helpful?
  - What did you think about the timing of the reminders? The frequency of them?

As a secondary aim, we will also assess intervention efficacy through the assessment of each of the sedentary behavior outcomes (total time, number of breaks). Objective methods of measurement are preferred to avoid biases and recall problems associated with self-reporting.

Data Analysis plan: For the qualitative analysis, a combination of recursive abstraction and content analysis will be used. Recursive abstraction involves generating iterative summaries of the verbal transcripts which are further abstracted to more general summaries. Content analysis will be used to quantify and rank the relative dominance of themes that emerge as most important from the abstraction process. Trustworthiness of the data will be evaluated using member validation of the verbal and text-based summaries derived from the interviews. Questionnaire data will be aggregated and presented using descriptive statistics (means, medians, proportions). Data on the primary intervention target, collected using the activPAL, will be processed using established protocols and presented using descriptive statistics. In the exploratory component of the study, within- (difference from baseline to follow-up) and between-person (baseline – intervention #1, #2,) comparisons will be conducted using (repeated-measures) analysis of covariance, adjusted for appropriate confounding factors. Interpretation of the results will focus upon effect estimates due the high risk of type 2 error associated with small samples.

Focus Groups:
This pilot study was conducted to determine the acceptability and feasibility of selected personal, social and environmental strategies to reduce overall sitting time and increase the number of times participants stand up in a day as well as to assess whether existing and new measurement approaches can detect specific changes in sedentary behavior. Information gathered from the pilot is being used to inform a program grant proposal being submitted through the Department of Family and Preventive Medicine to the National Institutes on Aging. Three focus groups of 5-10 people will be conducted to: 1) learn more about the specific intervention strategies utilized in the pilot study and how they might be adapted for a larger intervention study, and 2) learn more about what features of a device are most important and essential to help participants change their sedentary behavior and to identify ideal interfaces that would allow participants to interact with their data in a meaningful way in a future study.

Sample general questions include:
- What did you think about the general process of sitting less when you tried it during the study?
- You received prompts to remember to take sitting breaks. In what ways were these reminders helpful or not helpful?
- Since taking part in the study, how has sitting less been going for you?

Sample questions related to existing devices include:
- What do you like about this device/its feedback? What do you not like? What would you change if you could?
- Would you be willing to wear a device like this? For how long?
- Would you find this helpful to take breaks from sitting or spend less time sitting?
- Would you anticipate any specific problems wearing this kind of device?
- What do you think about the information this device records and reports? Is it interesting to you? Is there other feedback you would want to get?
- Are you comfortable with the interface of this device?
- Would you want this feedback in a different way? (device other than smartphone? Computer?)

Testing Protocol:
The primary objective of the testing protocol is to better understand the accuracy of devices that are designed to measure sedentary behavior (i.e., sitting, standing, lying) in quantifying transitions from a seated position to a standing position. There is currently a lack of information in how well these devices capture sit to stand transitions, thus further investigation is needed through a more extensive testing protocol. This information will be utilized to determine the best device to use in future sedentary behavior interventions. Participants will be asked to participate in 5 brief testing protocols wearing one of the measurement devices (e.g., ActivPAL, lumoback) and perform behaviors that include variations of going from a seated position to standing, in specific time windows (e.g., up and down every 10 seconds).

The 5 separate tests include: 1) rapid stands (i.e., stand up as many times as possible in 1 minute) 2) one second test (i.e., up from chair every second); 3) two second test (i.e., up from chair every two seconds); 3) five second test; 4) ten second test; 5) ten second stands, one second sit.

10. HUMAN SUBJECTS

A total of 30 participants will be enrolled from UCSD or surrounding neighborhoods. We aim for half of the sample to be currently working. Potential study participants will provide verbal consent to be screened for eligibility over the telephone. Enrollment and informed consent will occur in-person. Participants will be sedentary, with 1/3 of the population being overweight or obese and 1/3 being of Hispanic origin.

Inclusion Criteria:

1. Males or females 50 -70 years of age
2. Able to attend 4 measurement visits with study staff in 3 consecutive weeks
3. Spend at least 8 hours per day sitting
4. Willing and able to wear study device for 21 days
5. Able to read and write in English
6. Able to provide written informed consent

Exclusion Criteria:

1. Do not sit for at least 8 hours per day
2. Unable to attend 4 visits
3. Diagnosis of serious chronic condition that would limit the ability to stand

Focus groups:
We expect to recruit 5-10 participants for 3 separate focus groups (for a total of 15-30), who have already completed the pilot study. All participants who agreed to be contacted by us in the future on their consent form will be contacted to see if they are interested in participating.
Inclusion Criteria:

- Must have completed the pilot study.
- Must be willing to attend a 2 hour session.

Testing protocol:
We expect to recruit 10 participants for the testing protocol. Following the initial testing protocol, previous participants may be asked to come back to complete the protocol an additional time wearing a different device.
Inclusion criteria:

- Be able to provide informed consent,
- Able to read and write in English,
- Be willing to wear measurement devices.
- Able to participate in a brief (approximately 20 minutes) testing protocol involving transitions from a chair into a standing position.

11. RECRUITMENT

A sample of 30 participants will be recruited from employees on the University campus and surrounding neighborhoods. We aim to recruit participants who sit for the majority of the day, thus will target office workers or those with self-reported sedentary lifestyles. Participants will be recruited through the UCSD flyers listserv and Craigslist advertisements, which we have utilized successfully for recruitment in previous research studies. Non-working older adults will be recruited through a direct marketing approach via local media outlets including radio advertisements, print newspaper advertisements, television news stories, online newsletters, websites, flyers, and local organizations/community centers. Interested individuals will be directed to contact the research office by phone for additional information about the study and to be screened by phone to determine if they meet the primary inclusion criteria of the study population. Verbal informed consent will be obtained from potential participants.

Focus group participants will be recruited from the list of study participants who agreed to be contacted in the future by our research staff. We have this information stored in our study database.

Testing protocol participants will be recruited from the UCSD flyers listserv and will be directed to contact the research office by phone for additional information and to be screened by phone to determine if they meet the inclusion criteria.

12. INFORMED CONSENT

Interested individuals will be directed to contact the research office by phone. Potential participants will have the study purpose, procedures, risks and benefits described to them. Verbal consent to participate will be obtained at the time of the telephone screening. We are requesting a waiver of written consent for the telephone screening of potential subjects because the telephone screening presents no more than minimal risk of harm to subjects and involves no procedures for which written consent is normally required outside of the research context. Since we anticipate receiving many calls and the screening criterion are simple and few, it is more convenient and cost-efficient for participants to complete the screening by phone. We believe that a requirement for individuals to be screened in person (with no incentive offered) would serve as a deterrent to participation and limit the representation of our sample. Written consent will be obtained in person prior to beginning the study and participants will be given copies of the Experimental Subjects Bill of Rights. During the screening call, the study will be described in more detail and participant eligibility will be determined using the inclusion and exclusion criteria (see above). After initial screening, those eligible and interested will be invited to schedule an appointment at the research office to receive a more in-depth orientation to the study protocol and provide informed consent. At the orientation session, research staff will describe the study in more detail, explain the risks and benefits of participating, and answer any questions. Interested participants will then be given a consent form to read as well as time to ask further questions. Written informed consent will be obtained before any data are collected. A study investigator will ensure that consents are signed prior to data collection.

Focus group participants will complete an informed consent form at the beginning of the focus group, prior to any data collection.

Testing protocol participants will complete an informed consent form at the beginning of the testing period, prior to any testing procedures or data collection.

13. ALTERNATIVES TO STUDY PARTICIPATION

The alternative to study participation is to not participate.

14. POTENTIAL RISKS

Risks to participants in this study are considered minimal. The goal of the intervention is to reduce sitting time, whether that means moving more or simply standing up. Participants are unlikely to experience harms greater than those encountered in normal life because standing and walking are typical daily activities. Extended periods of standing can place additional load on the circulatory system and joints, which may be problematic for some people. Participants that have conditions that could be worsened by standing will be screened out. Further, older adults may experience dizziness when standing up.

There is a possibility of some physical, psychological, social, economic, and legal risk.

- Physical risks include a chance of physical injury if a device bruises the participant as a result of an incidental fall.
- Participants may also experience fatigue and muscle soreness during the course of the testing protocol. Psychological risks include embarrassment or anxiety over the type of questions being asked in a self-report survey.

Social or economic risks include a possibility of being teased or ridiculed by peers for wearing the devices or being socially excluded; or as a result of a breach of confidentiality of personally identifiable data.

15. RISK MANAGEMENT PROCEDURES AND ADEQUACY OF RESOURCES

Although all risks are considered minimal, psychological or social risk as a result of wearing a sensor will be managed by informing participants that they may interrupt or discontinue their involvement at any time. Participants will also be told that they can take off the sensor at any time. Participants will be guided to gradually increase their standing time and to place hands on a table, chair or wall for support when transitioning to a standing position. Finally, confidentiality and data security procedures will be explained to participants (these are described in more detail below).

Physical, psychological, social, or economic risk as a result of a breach in confidentiality will be managed by (a) using devices that cannot be downloaded by participants, only authorized members of the research team. Specifically, we keep informed consent statements and participant data in separate locked files cabinets so that individuals are not easily connected to the study results. All sensor data will be stored on a firewall and password-protected project server at the University of California, San Diego. To reduce the risk to (and fear of) confidentiality, all subject records and data will stripped of individual identifiers following data collection. We will assign each person a study ID and all records will be coded with the study ID rather than personal identifiers. The code that links the study ID and the name will be stored in a separate place than the data file until all of the measurements are complete. At that point, the personal information will be destroyed and all analyses will be conducted with the data set that has no personal identifiers. All data will be kept in locked cabinets at the study office, accessible only by investigators and project staff. Participants will also be told about the confidentiality procedures and that they have the right to refuse to answer questions or to terminate their participation in the study at any time without prejudice. Finally, participant data will not be sold or exchanged with anyone and data sharing as per NIH requirements will be performed within strict protocol-driven procedural guidelines. The PI will assure that all human protections standards are met.

This study involves no experimental procedures or behavioral manipulations, only device-based behavioral monitoring of a participant's usual daily routines. All study procedures and subject involvement involve minimal risk. Data monitoring procedures are therefore commensurate with the risks. Because of the minimal risk, the data and safety monitoring (DSM) plan for this study focuses on close monitoring by the Principal Investigator (PI) with prompt reporting of serious adverse events (including disclosure events) to the university Institutional Review Board (IRB) and, if applicable, NIH program officer staff. Data for monitoring and safety will come from self-reports from participants, or direct observations from research staff. The project coordinator will be responsible for assembling the data and producing these reports, as well as assuring that all relevant parties obtain copies of these reports. The reports will include information about subject accrual (adherence to protocol regarding demographics, inclusion/exclusion); adverse event rates; as well as statistical power implications of drop outs and missing data.

Testing Protocol;
Participants will be instructed that if they become tired or fatigued at any time during the testing period, they should immediately sit down to rest and recover. The testing period should take approximately 20 minutes to complete and participants will be instructed to go at their own pace and to take as many breaks as necessary between tests.

16. PRIVACY AND CONFIDENTIALITY CONSIDERATIONS INCLUDING DATA ACCESS AND MANAGEMENT

We have established an extensive protocol to protect participants' privacy. We will keep informed consent statements and participant data in separate locked files cabinets so that individuals are not easily connected to the study results. All sensor data will be stored on a firewall and password-protected project server at the University of California, San Diego. To reduce the risk to (and fear of) confidentiality, no identifying data will be collected in the field, only in a secure research laboratory. All subject records and data will be stripped of individual identifiers following data collection. We will assign each person a study ID and all records will be coded with the study ID rather than personal identifiers. The code that links the study ID and the name will be stored in a separate place than the data file until all of the measurements are complete. At that point, the personal information will be destroyed and all analyses will be conducted with the data set that has no personal identifiers. All data will be kept in locked cabinets at the study office, accessible only by investigators and project staff. Participants will also be told about the confidentiality procedures and that they have the right to refuse to answer questions or to terminate their participation in the study at any time without prejudice. Finally, participant data will not be sold or exchanged with anyone and data sharing as per NIH requirements will be performed within strict protocol-driven procedural guidelines. The PI will assure that all human protections standards are met.

17. POTENTIAL BENEFITS

Sedentary behavior (SB) is a health risk, independent of moderate-to-vigorous physical activity (PA) because it is related to numerous cancers, cardiovascular disease, metabolic syndrome, incident hypertension, and Type 2 diabetes. This research has the potential to greatly improve our understanding of feasible and effective intervention strategies, as well as improve our ability to measure sedentary behaviors that are known to be related to health. This is especially important in an aging population that could greatly benefit from reduced sedentary time.

By participating in this study, participants will gain knowledge of their own sedentary behavior habits and ways to improve them. There is a potential for multiple health benefits to occur in all participating individuals. In this age population in this setting, health is likely to be on the decline, decreased sedentary time may help prevent this decline.

18. RISK/BENEFIT RATIO

The risks of the current study are reasonable in that they rarely occur and precautions will be taken to minimize risks. The known risks to participants are outweighed by the potential benefit they may experience to their health.

19. EXPENSE TO PARTICIPANT

There will be no expense to the subject other than time. Participants will not be financially liable for the cost of the replacement of the measurement device, should it be lost or damaged.

20. COMPENSATION FOR PARTICIPATION

Participants will receive $50 after completing each week of the intervention. The incentive is dependent upon participants providing the minimum amount of usable data. Therefore, incentives will be paid only if participants wear the device for a minimum or 5 days per week. Payments will be made by Scrip, which is a preprinted check that can be cashed at their bank.
Participants who wear the device for the baseline week but who do not engage in at least 8 hours of sitting per day will not be enrolled in the study. Additionally, participants who do not wear the device in the first week for at least 5 days will not be enrolled. In both cases, they will receive $25 for their time and will not be asked to complete any additional tasks.

Focus group participants will receive $20 scrip for their participation.
Testing protocol participants will not receive any compensation; however water and light refreshments will be available throughout the course of the testing period.

21. PRIVILEGES/CERTIFICATIONS/LICENSES AND RESEARCH TEAM RESPONSIBILITIES

<u>Jacqueline Kerr, PhD, Principal Investigator</u> Dr. Kerr is assistant professor in the Department of Family and Preventive Medicine at the University of California, San Diego in the Center for Wireless and Population Health Systems at CALIT2. She also has adjunct professor status in the Departments of Psychology and Public Health at SDSU. She has received direction and mentoring from Drs. Sallis and Patrick, both highly successful investigators. She received her PhD from the University of Birmingham, England. Since her arrival in the US in 2004, Dr. Kerr has worked on 20 grants, as PI on six. Building upon existing intervention programs, Dr Kerr is well placed to conduct the pilot studies outlined herein. She has an established track record of converting small grants into larger funded projects. Further, in her existing MIPARC R01 study we have already developed educational materials aimed at reducing sedentary behavior which we can employ in this pilot. The sedentary component in MIPARC was only one session among many focusing on physical activity. We learned from this experience the difficulty in continuous self-monitoring of sedentary behaviors, and that individuals need to find prompting mechanisms that work realistically with their daily lives. In addition, the behavioral and theoretical framework for modifying sedentary behavior is more similar to stair climbing interventions than physical activity more broadly. Taking the elevator is an automatic behavior that requires real time prompting to change and is influenced by our environment and social norms. Dr Kerr's PhD thesis focused on stair climbing, supporting her ability to transition to this new behavioral challenge.

Andrew Atkin, PhD is a post-doctoral Career Development Fellow at the Centre for Diet and Activity Research (CEDAR), University of Cambridge. He has collected data on sedentary behaviour using both objective and self-report methods and contributed towards the development and evaluation of interventions to promote physical activity and reduce sedentary behaviour. He has published peer-reviewed articles on the measurement, health outcomes and determinants of sedentary behaviour. Alongside Dr Kerr, he jointly conceived the proposed pilot studies and contributed to writing the funding application. Dr Atkin's role in the proposed study will be to advise on study methodology and critically review study materials.

Jordan Carlson, PhD is an NIH T32 Postdoctoral Fellow in cardiovascular disease epidemiology. He is working on multiple projects investigating how policies and environments impact physical activity, sedentary time and cardiovascular health outcomes. He has expertise in measurement, including working with GPS and accelerometer data. In the present study, he will assist with data processing and analyses of accelerometer and inclinometer device measures. This will involve deriving metrics from the devices that can be used to assess protocol compliance and study outcomes.

Gina Merchant, MA is a Doctoral student in the UCSD/SDSU Joint doctoral program, focusing on Health Behavior. She will assist with the development of the semi-structured interviews.

Katie Crist, MPH, Project Manager is currently the coordinator of the MIPARC study in 10 retirement communities. She will have oversight for all parts of the study, ensuring staffing training and material needs are met. Ms. Crist will be responsible for recruitment and will act as a liaison between participants and staff. She will be responsible for the IRB documentation, quality control systems, and adverse event monitoring.

Khalisa Bolling, MPH, Health Educator is an experienced health educator who currently works on a physical activity study in older adults that includes a sedentary behavior intervention. Under the supervision of Dr. Kerr, she will deliver the educational session introducing pilot participants to sedentary behavior. She will also meet with participants each week to conduct interviews about participant's experience with the previously assigned condition and instruct them on the next sedentary strategy and goal.

Michelle Black, is currently pursuing a PhD in Public Health through the Joint Doctoral Program (JDP) at San Diego State University (SDSU) and UCSD. She holds a bachelor's degree in cognitive science and psychology. She will assist with the development and delivery of the interviews.

Brittany Lewars, Eileen Johnson, Annelise Brochier, Recruitment and measurement Research Assistants, All 3 RAs have experience with physical activity data collection and instructing participants in the use of these devices in the TREC study. The RAs will be responsible for assisting with participant recruitment, responding to participant inquiries about the study, conducting device distribution and collection visits, instructing participants in how to wear the devices and answering any questions or concerns, obtaining informed consent from participants and assisting the Health Educator with material development. They will perform data entry and transcription of the semi-structured interviews.

Claudia Pena is a Research Assistant who will help with the testing of the ActivPal device as well as summarizing data from the focus groups.

Lu Wang, MS Statistics, Statistician Assistant will be responsible for cleaning and coding study data. Ms Wang has experience with physical activity sensor data and is proficient in new statistical techniques in R. She will conduct all descriptive statistics and assist investigators with creating tables and figure to present the data. For the exploratory aims, she will use (repeated-measures) analysis of covariance to assess within- (difference from baseline to follow-up) and between-person (baseline – intervention #1, #2, #3) comparisons, adjusted for appropriate confounding factors.

Nadir Webil, PhD is a Research Scientist in the Department of Computer Science and Engineering at UCSD. His work is in Human-Centered Computing; investigating tools and technologies that can be used to measure or promote health behaviors. He will assist with designing and running the focus group investigating feasibility and design of a tool to measure sedentary behavior.

Dori Rosenberg, PhD, MPH is an Assistant Scientific Investigator at GHRI and an Affiliate Assistant Professor in the Department of Health Services at the University of Washington's School of Public Health. Her background is in behavioral science through her advanced degrees in clinical psychology and public health. She has specific expertise in promoting physical activity and sedentary behavior change among various populations including youth, older adults, and people with chronic medical conditions. Dr. Rosenberg is a co-Investigator on Dr. Kerr's MIPARC study as she helped develop all intervention materials and measurement assessments for the HAPS pilot program, which informed MIPARC. She will assist with the development and analyses of the focus groups.

22. BIBLIOGRAPHY

[1] Katzmarzyk PT. Physical activity, sedentary behavior, and health: Paradigm paralysis or paradigm shift? *Diabetes* 2010;**59**:2717-25.

[2] Owen N, Leslie E, Salmon J, Fotheringham MJ. Environmental determinants of physical activity and sedentary behavior. *Exerc Sport Sci Rev* 2000;**28**:153-58.

[3] Sedentary Behaviour Research Network. Letter to the Editor: Standardized use of the terms "sedentary" and "sedentary behaviours". *Appl Physiol Nutr Metab* 2012;**37**:540-2.

[4] Matthews CE, Chen KY, Freedson PS, *et al.* Amount of time spent in sedentary behaviors in the United States, 2003-2004. *Am J Epidemiol* 2008;**167**:875-81.

[5] Dunstan DW, Barr EL, Healy GN, *et al.* Television viewing time and mortality: The Australian Diabetes, Obesity and Lifestyle Study (AusDiab). *Circulation* 2010;**121**:384-91.

[6] Dunstan DW, Salmon J, Healy GN, *et al.* Association of television viewing with fasting and 2-h postchallenge plasma glucose levels in adults without diagnosed diabetes. *Diabetes Care* 2007;**30**:516-22.

[7] Grontved A, Hu FB. Television viewing and risk of type 2 diabetes, cardiovascular disease, and all-cause mortality: a meta-analysis. *JAMA* 2011 Jun 15;**305**:2448-55.

[8] Hamer M, Stamatakis E, Mishra GD. Television- and Screen-Based Activity and Mental Well-Being in Adults. *Am J Prev Med* 2010;**38**:375-80.

[9] Healy GN, Dunstan DW, Salmon J, Shaw JE, Zimmet PZ, Owen N. Television time and continuous metabolic risk in physically active adults. *Med Sci Sports Exerc* 2008;**40**:639-45.

[10] Proper KI, Singh AS, van Mechelen W, Chinapaw MJ. Sedentary behaviors and health outcomes among adults: A systematic review of prospective studies. *Am J Prev Med* 2011;**40**:174-82.

[11] Wijndaele K, Brage S, Besson H, *et al.* Television viewing time independently predicts all-cause and cardiovascular mortality: The EPIC Norfolk Study. *IntJ Epidemiol* 2010.

[12] Wijndaele K, Lynch BM, Owen N, Dunstan DW, Sharp S, Aitken JF. Television viewing time and weight gain in colorectal cancer survivors: A prospective population-based study. *Cancer Causes Control* 2009;**20**:1355-62.

[13] Hamilton MT, Hamilton DG, Zderic TW. Exercise physiology versus inactivity physiology: An essential concept for understanding lipoprotein lipase regulation. *Exerc Sport Sci Rev* 2004;**32**:161-66.

[14] Hamilton MT, Hamilton DG, Zderic TW. Role of low energy expenditure and sitting in obesity, metabolic syndrome, type 2 diabetes, and cardiovascular disease. *Diabetes* 2007;**56**:2655-67.

[15] Atkin AJ, Gorely T, Clemes SA, *et al.* Methods of Measurement in epidemiology: Sedentary Behaviour. *Int J*

Epidemiol 2012 Oct;**41**:1460-71.

[16] Wareham NJ, Rennie KL. The assessment of physical activity in individuals and populations: Why try to be more precise about how physical activity is assessed? *Int J Obes Relat Metab Disord* 1998;**22 Suppl 2**:S30-8.

[17] Baranowski T, Anderson C, Carmack C. Mediating variable framework in physical activity interventions. How are we doing? How might we do better? *Am J Prev Med* 1998 Nov;**15**:266-97.

[18] Sallis JF, Owen N. Physical activity and behavioural medicine: Thousand Oaks, CA: Sage 1999.

[19] Rhodes RE, Mark RS, Temmel CP. Adult sedentary behavior: a systematic review. *Am J Prev Med* 2011 Mar;**42**:e3-28.

[20] Marteau TM, Hollands GJ, Fletcher PC. Changing human behavior to prevent disease: the importance of targeting automatic processes. *Science* 2012 Sep 21;**337**:1492-5.

[21] Conroy DE, Maher JP, Elavsky S, Hyde AL, Doerksen SE. Sedentary Behavior as a Daily Process Regulated by Habits and Intentions. *Health Psychol* 2013 Mar 11.

[22] Owen N, Sugiyama T, Eakin EE, Gardiner PA, Tremblay MS, Sallis JF. Adults' sedentary behavior determinants and interventions. *Am J Prev Med* 2011 Aug;**41**:189-96.

[23] Heath GW, Parra DC, Sarmiento OL, *et al*. Evidence-based intervention in physical activity: lessons from around the world. *Lancet* 2012 Jul 21;**380**:272-81.

[24] Gilson ND, Suppini A, Ryde GC, Brown HE, Brown WJ. Does the use of standing 'hot' desks change sedentary work time in an open plan office? *Prev Med* 2012 Jan;**54**:65-7.

[25] Koepp GA, Manohar CU, McCrady-Spitzer SK, *et al*. Treadmill desks: A one-year prospective trial. *Obesity (Silver Spring, Md* 2013 Nov 6.

[26] Fogg BJ. Stanford Persuasive Technology Lab. 2013 Accessed 25/03/2013 [cited; Available from: http://captology.stanford.edu/

[27] Kozey-Keadle S, Libertine A, Lyden K, Staudenmayer J, Freedson P. Validation of wearable monitors for assessing sedentary behavior. *Med Sci Sports Exerc* 2011;**43**:1561-67.

[28] Kerr J, Marshall SJ, Godbole S, *et al*. Using the SenseCam to Improve Classifications of Sedentary Behavior in Free-Living Settings. *Am J Prev Med* 2013 Mar;**44**:290-6.

23. FUNDING SUPPORT FOR THIS STUDY

Department of Family and Preventive Medicine Pilot Study Award – Index #CFMJKPS

24. BIOLOGICAL MATERIALS TRANSFER AGREEMENT

N/A

25. INVESTIGATIONAL DRUG FACT SHEET AND IND/IDE HOLDER

N/A

26. IMPACT ON STAFF

N/A

**27. CONFLICT OF INTEREST**
There are no conflicts of interest.
**28. SUPPLEMENTAL INSTRUCTIONS FOR CANCER-RELATED STUDIES**
N/A
**29. OTHER APPROVALS/REGULATED MATERIALS**
N/A
**30. PROCEDURES FOR SURROGATE CONSENT AND/OR DECISIONAL CAPACITY ASSESSMENT**
N/A

Version date: May 11, 2011

University of California, San Diego
Consent to Act as a Research Subject in a Sedentary Behavior Intervention

Research Study: Decreasing Sitting Time

Jacqueline Kerr, PhD, has been funded by the Department of Family and Preventive Medicine to conduct this research study to assess sedentary behavior in older adults. Before you decide to participate, it is important that you read the following information and ask as many questions as necessary to be sure you understand what you will be asked to do.

Purpose of the Study: Older adults are the age group with the highest amount of sedentary time (time spent sitting or reclining) and the lowest amount of physical activity. New evidence links high amounts of sedentary time with unhealthy effects among older adults such as diabetes, cancer, mortality, obesity, and poor cholesterol ratio. The purpose of this study is to see whether we can develop a program to help people reduce their sitting time and to see if participants are satisfied with the program.

You are being asked to participate in a research study that lasts for 3 weeks. There will be approximately 30 people from the San Diego area in this study.

To be eligible for this study you have to meet the following criteria:

1. Be 50-70 years of age
1. Spend at least 8 hours per day sitting
2. Able to attend 4 measurement visits with study staff over the course of the next 3 weeks
3. Willing and able to wear study device 24 hours per day for 21 days
4. Able to read and write in English
5. Able to provide written informed consent
6. Do not have a serious chronic condition that would limit your ability to stand

Description of the Study
There are 2 groups in this study; one focused on decreasing the amount of time you spend sitting and one focused on increasing the number of times you stand up each day. You will be randomly assigned to one of these groups.

If you join the study, we will ask you to:

1. **Complete 4 visits with our research staff that will last approximately 1-1$^{1/2}$ hours each.** You will learn about sedentary behavior and will talk with a health educator to figure out ways to try to reduce your sitting time or increase the number of times you stand up each day.
2. **Wear a small activity monitor called the activPAL.** The activPAL is worn on the front of your thigh, underneath your clothing. We will ask you to wear it all day and night for the 3 weeks of the study as you go about your normal routine. This will allow us to see your sitting, standing, and moving habits without you having to write them down.
3. **Complete a short survey at the first and last visit.** The surveys will ask you questions about your health, perceptions and daily activities.
4. **Participate in interviews at each visit.** We would like your feedback each week about your experience with the program. We would like to audio record the discussion for research purposes only. You can still participate in the interview even if you do not agree to have the conversation recorded.

   ___Yes, you agree to have the interviews recorded.
   ___No, you do not agree to have the interviews recorded.

5. **Complete a brief daily activity log.** This log will track when you wake up, go to sleep and, if you work, when you arrive at and leave your job.
6. **Utilize tools and strategies provided to you to attempt to reduce your sedentary time.** These strategies can include: working at a standing desk, setting reminders or timers to take standing breaks, standing in situations where you may typically sit (i.e. meetings or while on the phone).
7. **Receive study reminders via text, email or telephone.**

<u>What is Experimental in this Study</u>
Two methods for reducing overall sedentary time are being tested. Data from the two groups will be compared.

<u>Risks or Discomforts</u>
The risks involved in this study are minimal. You might feel uncomfortable answering some study questions. You may skip any questions you don't want to answer. You may experience mild muscle soreness, stiffness, and/or fatigue initially as you increase the time you spend standing each day. These should diminish over time as your body becomes used to standing and moving more. You may experience local skin irritation from the activPAL's mild adhesive. You also might fall from

standing more often. If you choose to increase your physical activity level, there is a chance you could injure yourself. Although we think it is unlikely that you will experience any physical harm from this study, it's important to seek immediate health care attention if you experience chest pain, lightheadedness, an injury, or symptoms that concern you.
It's possible that someone other than the researchers could find out you were in the study or see your private study information. The steps we take to keep this from happening are described below. Since this in an investigational study, there may also be some unknown risks that are currently unforeseeable.

Confidentiality
Research records will be kept confidential to the extent provided by law. All responses and data collected will be kept confidential within the research team and your data will be stored appropriately in locked cabinets and on a secure computer server. All information that is stored on our secure computer will be identified by subject ID number only. Results of this study may be reported in scientific journals, meetings, and news media. None of these reports will use your name or use data that can point to any person who took part in the study. Strict security measures will be taken to insure that none of these procedures results in release of any information you provide us. Research records may be reviewed by the UCSD Institutional Review Board.
You give your permission to the researchers to store your data for use in secondary analyses that have not yet been specifically planned at this time. At a later date, if you decide that you do not want the data collected from you to be used for future research, you may inform Dr. Kerr, who will use her best efforts to stop any additional analyses.

Benefits
By participating in this study, you will gain knowledge of your own sedentary behavior habits and learn ways you might improve them. This is especially important in an aging population that could greatly benefit from reduced sedentary time. We cannot, however, guarantee that you will benefit from being in this study. We do hope the results of this study will help ongoing and future work in reducing sedentary (sitting) time.

Payment for Participation
You will receive a $50 incentive after wearing the devices for 7 days, trying the strategies to sit less, and completing a visit with our staff each week. The incentive will total $150 for completing all 3 weeks of the study. If you do not meet the eligibility requirement of sitting for at least 8 hours per day or were unable to wear

the device for 7 days during the first week, you will receive $25 for your time but will not be enrolled in the study. You will be paid via a preprinted check that can be cashed at any bank. No form of ID is required to cash the check.
There will be no cost to you other than your time. You will not be financially liable for the cost of the replacement of the measurement device should it be lost or damaged.

Voluntary Nature of Participation
Participation in this study is entirely voluntary. You may refuse to participate or withdraw at any time. Your decision of whether or not to participate will not jeopardize your future relations with the University of California San Diego or benefits to which you are otherwise entitled.

You may be withdrawn from the study if the Principal Investigator believes that it is in your best interest. You will be withdrawn from the study if the activity device shows that you do not spend at least 8 hours per day sitting or that you did not wear the device for 7 days.

Alternatives to Participation
The alternative to participating in this research project is to choose not to participate.

Future Contact:
We may want to contact you again at some point in the future to get your feedback on what you liked or didn't like about this study or to let you know about future studies that you may like to participate in. You do not have to agree to being contacted again.

Do you agree to be contacted in the future by study staff?
___Yes, you agree to be contacted in the future by study staff.
___No, you do not agree to be contacted in the future by study staff.

Care If Harmed
If you are injured as a direct result of an activity performed by a UCSD employee, the University of California will provide any medical care needed to treat those injuries. The University of California will not provide any other form of compensation if you are injured. You may call the UCSD Human Research Protections Program office at (858) 657-5100 for more information about this, to inquire about your rights as a research subject, or to report research-related problems. You may also reach the HRPP office by mail at:

University of California, San Diego
Human Research Protections Program
9500 Gilman Drive, Mail Code 0052
La Jolla, California, 92093-0052

Procedures for Termination
If you decide to withdraw from this study, you will be required to notify a member of the research team and return the devices if they are still in your possession.

Questions about the Study: Dr. Kerr or ________________________has explained this study to you and answered your questions. If you have questions later about the research, you may contact Katie Crist, Project Manager, at (858) 534-9306.

Agreement: The University of California, San Diego Institutional Review Board has approved this consent form as designated by the Board's stamp. The consent form must be reviewed annually and expires on the date indicated on the stamp.

Your signature below indicates that you have read the information in this document and have had a chance to ask any questions you have about the study. Your signature also indicates that you agree to be in the study and have been told that you can change your mind and withdraw your consent to participate at any time. A copy of this consent form will be given to you.

____
Name of Participant (please print)

____ ______________
Signature Date

____ ______________
Witness Date
